# Response: Commentary: The Human Default Consciousness and Its Disruption: Insights From an EEG Study of Buddhist Jhāna Meditation

**DOI:** 10.3389/fnhum.2020.00020

**Published:** 2020-02-05

**Authors:** Paul A. Dennison

**Affiliations:** Independent Researcher, London, United Kingdom

**Keywords:** meditation, EEG, Jhana, consciouness, infraslow activity, spindles, spike-waves

I will deal with Fell et al.'s (2019) comments in order.

The meditation spindles and slow waves described in my paper, Dennison (2019), are reminiscent, but only reminiscent of sleep EEG. The methodology was to analyse similarities and differences to determine whether they represent a sub-group of sleep activity, or different mechanisms.

## Spindles

Meditation spindles were indeed found to be sufficiently different to both slow and fast sleep spindles to indicate a different mechanism. The authors appear to have misread or misunderstood this methodology and their comment, “he [Dennison] still interprets his observations as related to sleep-like activity” is puzzling, and their further comment “he nonetheless interprets them to reflect the thalamocortical dynamics characteristic of sleep spindles” is both inaccurate and misleading. My argument was that thalamo-cortical networks are likely to be involved since the thalamus is generally regarded as the only brain region capable via the reticular nuclei of triggering spindle and indeed spike-wave behavior (Avanzini et al., 2000). Also, since spindles whether in meditation, sleep, attentional distraction, or anesthesia share a common theme of disruption to attention, it is reasonable to assume that all those modalities involve thalamo-cortical mechanisms, albeit with different network characteristics.

**Figure 1:**
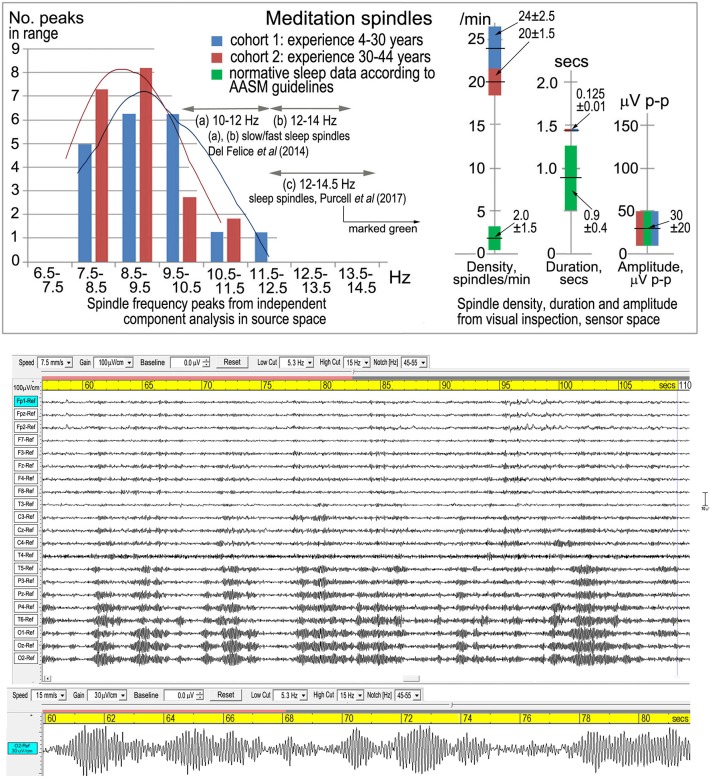
Meditation spindles. Top panel from Dennison (2019), Figure 2, shows spindle density, duration, amplitude and frequency in sensor space from visual inspection according to American Academy of Sleep Medicine guidelines [American Academy of Sleep Medicine (AASM), 2017]. The lower panel is one of three examples from Dennison (2019), Figure 1, showing the symmetrical waxing and waning wave-packet-like morphology of spindles.

Fell et al. (2019) further state that meditation spindle spectral peaks “overlap perfectly” with typical alpha activity, which is patently incorrect (Figure 1, upper panel): there is a limited overlap with the low alpha band, and the meditation spectral peaks at 8.63 and 9.20 Hz are significantly lower than the central alpha peak of 10.00 Hz.

Their comment that “alpha activity is typical for waking rest” is of course correct, but their continuation “and like sleep spindles, shows waxing and waning activity patterns” is misleading. The term “spindles” describes a *defined*, recognizable and quantifiable *symmetrical* waxing and waning whether referring to stage-2 sleep, attentional distraction, early-stage anesthesia, or in this case meditation. The characteristic wave-packet-like morphology is shown in the lower panel of Figure 1.

My hypothesis that meditation spindles represent a disruption of alpha activity into spindle-like behavior is not unrelated to other situations of attentional distraction, except that in meditation the frequency is progressively lowered into the theta band as meditation experience increases (Figure 1, upper panel; two cohorts). The analysis in source space supports attentional distraction of the dorsal and ventral attention streams, consistent with Buddhist theories of jhāna meditation.

## Infraslow Waves (ISWs)

Similarly, meditational ISWs differ significantly from slow waves in sleep, indicating a different mechanism. Fell et al. (2019) comment “the author acknowledges this difference, but nonetheless relates this phenomenon to sleep-like activity” is again very puzzling.

I am astonished at Fell et al.'s (2019) suggestion that these rhythmic ISWs might be sweat artifacts, no doubt familiar to the authors through working in polysomnography and nighttime epilepsy monitoring. Sweat artifacts are easily recognized (and remedied) and invariably caused by subjects becoming overheated, often accompanied by disturbed electrode connectivity through tossing and turning during sleep. In contrast, meditation subjects are recorded in spacious well-ventilated rooms, wear light clothing, are relaxed and certainly not overheated. Impedances are carefully monitored before and after recordings and at least once during recording to confirm electrode contact integrity. The fact that meditational ISW characteristics are consistent across subjects, some re-recorded after 1–3 years, with different amplifier and headcap combinations, supports the integrity of the findings. Furthermore, the bottom panels of Figures 4 and 5 in Dennison (2019) show examples of rapid onset of powerful ISWs shortly after the cue to “start meditation”; most unlikely in my view to coincide with a sudden onset of acute sweating.

Fell et al. (2019) also suggest, dismissively, that meditational ISWs might be part of already “established phenomena,” and cite Watson (2018). In fact, Watson notes that ISWs are “a relatively understudied phenomenon,” with many questions unanswered as to their significance and origin, which in the absence of “causal experiments” may remain unanswered. Their occurrence in jhāna meditation is a unique and as far as I am aware first example of a causal situation, where meditators intentionally withdraw attention from default consciousness activity. As ISWs develop, analysis in source space demonstrates a shift in network activity toward a vertical axis, suggestive of a deep brain-body metabolic integration. They occur as meditation deepens beyond the more common spindle activity, and appear to correspond to the stage of high energization described in the jhāna literature. None of this is acknowledged by Fell et al. (2019).

## Spike-Wave Activity

Spike-wave activity is an area where, as epilepsy researchers, the authors might have been expected to make a constructive contribution. They begin by pointing out the differences between meditational spike-waves and those seen in epilepsy, implying that this is something I have not recognized. In fact this is the heart of my discussion, precisely that meditational spike-waves differ significantly from those in epilepsy. The frequency of the spike component varies across subjects unlike the more fixed ~3.5 Hz in absence epilepsy, and rather than the simple wave component between spikes in epilepsy, meditational spike-waves show pronounced harmonic activity (Table 4, Dennison, 2019). This feature and the analysis in source space are ignored by the authors, as well as potential implications of harmonic activity for understanding scaled frequency structure of cortical activity.

## Epileptiform Activity

The two examples in Figure 11 were included to complete an overview, while acknowledging this area is at an early stage of analysis. I acknowledged the complication of muscle artifacts during the clonic phase of this willed activity, and the authors are dismissive that any inferences can be made regarding cortical activity. The upper example however clearly shows cortical activity both in electrode space as well as source space, as described in the paper. The lower example of more pronounced clonic activity was included to illustrate exactly the point (Fell et al., 2019) mention of the difficulty of separating muscle artifacts from cortical activity.

## Summary

The authors appear to have misread or misunderstood the methodology and detailed analysis and discussion in the paper. Themes in observed EEG and underlying cortical sources correspond well with Buddhist theories of jhāna, and have intriguing resonances with emerging theories around free energy and active inference, as parallel formulations of what I refer to as our default consciousness. Rather than an opportunity for a constructive dialogue, I find their approach patronizing, simplistic and dismissive, including their use of “dubious” in referring to my paper. Rather than responding in the same vein, I hope my verdict is rather more playful: D^−^, null points.

## Author Contributions

PD is entirely responsible for the content of this Response.

### Conflict of Interest

The author declares that the research was conducted in the absence of any commercial or financial relationships that could be construed as a potential conflict of interest.
